# Rabbitpox in New Zealand White Rabbits: A Therapeutic Model for Evaluation of Poxvirus Medical Countermeasures Under the FDA Animal Rule

**DOI:** 10.3389/fcimb.2018.00356

**Published:** 2018-10-05

**Authors:** Mark R. Perry, Richard Warren, Michael Merchlinsky, Christopher Houchens, James V. Rogers

**Affiliations:** ^1^Battelle, Biomedical Research Center, West Jefferson, OH, United States; ^2^U.S. Department of Health and Human Services, Biomedical Advanced Research and Development Authority, Washington, DC, United States; ^3^BioMotiv, Cleveland, OH, United States

**Keywords:** rabbitpox virus, poxvirus, rabbit, animal model, FDA animal rule

## Abstract

The elimination of smallpox as an endemic disease and the obvious ethical problems with clinical challenge requires the efficacy evaluation of medical countermeasures against smallpox using the FDA Animal Rule. This approach requires the evaluation of antiviral efficacy in an animal model whose infection recapitulates the human disease sufficiently well enough to provide predictive value of countermeasure effectiveness. The narrow host range of variola virus meant that no other animal species was sufficiently susceptible to variola to manifest a disease with predictive value. To address this dilemma, the FDA, after a public forum with virologists in December 2011, suggested the development of two animal models infected with the cognate orthopoxvirus, intradermal infection of rabbits and intranasal infection of mice, to supplement the non-human primate models in use. In this manuscript, we describe the development of an intradermal challenge model of New Zealand White rabbits with rabbitpox virus (RPXV) for poxvirus countermeasure evaluation. Lethality of RPXV was demonstrated in both 9 and 16-weeks old rabbits with an LD_50_ < 10 PFU. The natural history of RPXV infection was documented in both ages of rabbits by monitoring the time to onset of abnormal values in clinical data at a lethal challenge of 300 PFU. All infected animals became viremic, developed a fever, exhibited weight loss, developed secondary lesions, and were euthanized after 7 or 8 days. The 16-weeks RPXV-infected animals exhibiting similar clinical signs with euthanasia applied about a day later than for 9-weeks old rabbits. For all animals, the first two unambiguous indicators of infection were detection of viral copies by quantitative polymerase chain reaction and fever at 2 and 3 days following challenge, respectively. These biomarkers provide clinically-relevant trigger(s) for initiating therapy. The major advantage for using 16-weeks NZW rabbits is that older rabbits were more robust and less subject to stress-induced death allowing more reproducible studies.

## Introduction

Variola major virus (VARV), the causative agent of smallpox, was declared eradicated by the World Health Organization in 1980. The elimination of this circulating episodic disease can be considered the greatest public health achievement in history as the disease is responsible for an estimated 300 to 500 million deaths in the 20th century; greater than the numbers killed in wars. However, the possibility that clandestine stocks of VARV still exist or a genetically-engineered strain could be generated and used as a weapon of bioterrorism in an unvaccinated and unprepared population (Chapman et al., 2010; Noyce et al., 2018) led the United States Government (USG) to reinvest in medical countermeasures that could be used during a naturally occurring or man-made smallpox outbreak. In 2014, discovery of vials containing viable VARV in a Bethesda, Maryland storage laboratory led to investigations by the Centers for Disease Control and Prevention (CDC), to identify potential accessibility of unknown quantities of live virus around the globe (Arita, 2014). It is not possible to study naturally-occurring smallpox disease in humans, since it is no longer endemic and it would be unethical to infect humans with smallpox. Therefore, evaluation of medical countermeasures for the treatment of smallpox requires a non-traditional approach.

Although product safety will be demonstrated using traditional clinical trials, the demonstration of efficacy, in the absence of clinical options, means the U.S. regulatory evaluation of putative medical countermeasures against smallpox will require use of the U.S. Food and Drug Administration (FDA) Animal Rule to demonstrate efficacy. Using the Animal Rule, the FDA may grant approval “based on adequate and well-controlled animal efficacy studies when the results of those studies establish that the drug is reasonably likely to produce clinical benefit in humans.” For an animal model to provide evidence supporting approval against smallpox, the surrogate disease in animals should provide predictive value with regard to the efficacy of the product when used in humans. The exquisite host range restriction of variola virus explains the absence of an animal reservoir and inability to infect animals, including non-human primates with the possible exception of orangutans (Fenner et al., 1988), at doses approximating the infectious dose in humans (Jahrling et al., 2004). The absence of a natural host for VARV infection means that product evaluation under the Animal Rule requires the use of surrogate models where the route of host infection and subsequent disease of the cognate orthopoxvirus is similar to smallpox in humans as to provide confidence of the medical countermeasure efficacy when used against smallpox. The model should possess high mortality at a low infectious dose, a disease course with stages and pathogenesis similar to smallpox in humans, and reproducible and unambiguous clinical biomarkers for countermeasure intervention. Since product efficacy will be measured in animals infected with a species-specific orthopoxvirus, conservation of target gene nucleotide sequence and protein structure should be demonstrated between the infecting virus and VARV as well as inhibitory activity against both viruses *in vitro*. In addition, efficacy assessment using the Animal Rule requires the sponsor to demonstrate that the mechanism of action is conserved between the animal and humans and the exposure levels of the drug at the proposed dose in humans exceeds the measured effective level in surrogate animal models.

The evaluation of medical countermeasures against smallpox requires the development of surrogate animal models of infection to evaluate product efficacy and dose so that one can confidently predict the efficacy of these countermeasures if they are used to treat human infections of smallpox. At the onset of the program to develop smallpox antivirals for the USG, the only animal model in use for regulatory evaluation of products was the monkeypox virus (MPXV) infection of non-human primates model (Earl et al., 2004). Both rhesus macaques and cynomolgus monkeys are susceptible to MPXV infection by a variety of routes (Chapman et al., 2010). However, there are limitations with these non-human primate models, including the requirement for high dose, limitations in group size inherent in the care and treatment of non-human primates, and the requirement by the FDA to compare the product exposure required for efficacy in multiple models that facilitate selection of the appropriate efficacious dose in humans. In order to determine additional appropriate animal models to use in the evaluation of smallpox countermeasures, the FDA Center for Drug Evaluation and Research (CDER) held an Antiviral Drugs Advisory Committee Meeting with drug sponsors and members of the scientific community to address smallpox concerns in December 2011. The resulting guidance specified that rabbits challenged by the intradermal route with rabbitpox virus (RPXV) and mice challenged by the intranasal route with ectromelia virus (ECTV) were acceptable models for further development as possible animal models to evaluate countermeasures.

In this manuscript, we describe the characterization and development of the intradermal infection of New Zealand White (NZW) rabbits with RPXV, which recapitulates several key features of smallpox infection in humans. Rabbitpox was initially reported in rabbit colonies housed at the Rockefeller Institute for Medical Research during the 1930's (Greene, 1933, 1934a,b, 1935). During the 1940's, an outbreak was also observed in a rabbit colony in Utrecht, The Netherlands. These studies used a purified stock derived from the RPXV Utrecht strain in the intradermal RPXV infection model using 9 and 16-weeks old NZW rabbits.

Previous studies using the 9-weeks old NZW rabbits characterizing the pathogenicity of intradermal RPXV infection and measuring the efficacy of poxvirus therapeutics (Adams et al., 2007; Denzler et al., 2011; Rice et al., 2011a; Trost et al., 2015) suggested this could serve as an excellent candidate for a regulatory model. These studies were not directly applicable under the Animal Rule for efficacy evaluation since the investigators initiated medical intervention based on time or subjective triggers not present in human smallpox infections (e.g., ear lesions). The development of smallpox antivirals by the USG is for use in a smallpox emergency as a therapeutic requires testing the efficacy of medical countermeasure therapeutic intervention based on exhibition of symptoms such as fever or lesions. For the rabbit model to provide a tool for evaluating potential smallpox antivirals, it should recapitulate the general pathology of smallpox infection and have biomarkers that correspond to those observed in humans that can serve as triggers for medical intervention. The course of the intradermal challenge disease, as described in Adams et al. (2007) is similar to that of smallpox in humans in which exposure to a relatively low dose results in an incubation phase without symptoms characterized by primary viral infection. This is followed by a fever, virus dissemination, and widespread systemic infection characterized by secondary lesions of the skin and mucocutaneous tissues. The major difference between smallpox and rabbitpox is that the disease progression in these small animals is compressed with a shorter incubation time (2 days in the rabbits compared to 7–17 days in humans), prodromal phase and resolution, with the mean time-to-death in rabbits of less than 10 days compared to humans where death generally occurs within 2 weeks after lesion formation.

There were two major aims of these studies; First, the course of RPXV disease progression was characterized by measuring the time to onset of statistically significant changes in body temperature, clinical observations, lesions, body weight, and the presence of circulating RPXV genomes by quantitative polymerase chain reaction (qPCR) in order to identify potential triggers for medical intervention, and; second the characterization of the course of infection was repeated in 16-weeks old rabbits to determine the robustness of a RPXV challenge model, demonstrating its utility in older, healthier 16-weeks NZW rabbits for the therapeutic evaluation of medical countermeasures.

## Materials and methods

### Rabbitpox virus

Rabbitpox Virus 090314-RPXV was derived from the Utrecht strain of RPXV (Lot#57590802) provided from ATCC. A master stock of the virus was generated by harvesting infected African Green Monkey Kidney Fibroblast Cells (CV-1) from 10 T-162 flasks, concentrating the cells by low seed centrifugation and lysis of the cell pellet with three freeze-thaw cycles. The lysed cell supernatant was used to infect 52 T-162 flasks of confluent CV-1 cells and after cell lysis, the infected cell supernatant was sonicated and overlaid on 36% sucrose and centrifuged at 48,000 g for 1 h. The pellet was suspended in phosphate-buffered saline (PBS). Viral tiers were determined by plaque assay similar to that previously described (Garver et al., 2016). Briefly, serial dilutions of samples were prepared in Eagle's Minimum Essential Medium (EMEM) containing 1% FBS and 0.1% gentamycin sulfate were plated onto confluent VERO E6 cell monolayers in triplicate. Viral suspensions (0.1 mL) were inoculated on the cell monolayers and adsorbed for 1 h at 37.0 ± 2°C and 5.0 ± 2% CO2 with rocking, followed by an overlay of 1.89% methyl cellulose in 2X EMEM with 10% FBS, non-essential amino acids, and 1% antibiotics. Following 72 h of incubation at 37.0 ± 2°C and 5.0 ± 2% CO2, the overlay media was removed, and the cells were stained with a 1.3 g/L crystal violet solution containing formalin. Plaques were enumerated manually using a light box and the average titer calculated. Results were reported in plaque forming units per milliliter (PFU/mL).

The Working Virus Stock was diluted in PBS to 8.75 × 10^6^ PFU/mL and distributed into aliquots. The virus stock was confirmed negative for mycoplasma by the Cambrex MycoAlert™ mycoplasma detection assay. Bacterial contamination and endotoxin levels were acceptable because they were below limits of detection (10 CFU/mL for bacteria and fungi and < 0.1 EU/mL for endotoxin by the Endosafe® Portable Test System).

### Test system

Specific pathogen-free NZW rabbits (*Oryctolagus cuniculus*) were obtained from Covance Research Products (Denver, PA). Thirty-six 9-weeks and thirty-six 16-weeks old rabbits (50% male and 50% female; *n* = 6 per age group) were used for the study to determine the LD_50_. Twenty-four 9-weeks and twenty-four 16-weeks rabbits (50% male and 50% female; *n* = 4 controls and *n* = 20 challenged per age group) were used on the natural history study of infection with 300 PFU of RPXV. The animals were maintained under an animal care and use program accredited by the Association for Assessment and Accreditation of Laboratory Animal Care (AAALAC) International and procedures approved by Battelle's Institutional Animal Care and Use Committee. Animals were maintained on a 12-h light/dark cycle with no twilight. Air temperature in animal rooms was maintained within a 61 to 72°F range, with relative humidity maintained between 30 and 70%. The animals were fed once daily (PMI Feeds, Inc.; St. Louis, MO), water was provided *ad libitum*, and housed individually. The study was conducted in a biosafety level 3 (BSL-3)/animal BSL-3 laboratory.

### Inoculations

For all animals, injection sites on both thighs were shaved on Study Day−1. On Study Day 0, target doses of the RPXV strain Utrecht were prepared by diluting the Working Virus Stock in Dulbecco's Phosphate Buffered saline (DPBS). The challenge sites were wiped with 70% isopropanol and allowed to air dry prior to inoculation. To measure the potency of the RPXV stock (LD_50_ experiments), dilutions were made to produce challenge titers of 1,000, 300, 81, 27, 9, and 3 PFU (100 μL in each thigh). For the natural history study, rabbits were challenged with 300 PFU for natural history studies to characterize disease progression. The challenge material was maintained on wet ice following preparation. Animals were anesthetized intramuscularly (IM) with a combination of ketamine (22–50 mg/kg) and xylazine (3–10 mg/kg) prior to challenge. For the natural history study, the challenge dose of 300 PFU was divided in half and all animals were inoculated by bilateral intradermal injections in each thigh region (100 μL in each thigh). For both studies, quantitative “back titration” plaque assays were performed on samples from the challenge dose dilution to confirm delivery of the virus. Sham-infected animals received DPBS injections (100 μL in each thigh) in the same manner as the RPXV-challenged animals (natural history study, only).

### Clinical data

Animals were observed twice daily (AM and PM) from the beginning of quarantine to the end of study (14 days after RPXV inoculation) to document inoculation site changes, pox lesion counts on the back, mouth, nose, and eye areas, and for adverse clinical signs of disease. All surviving animals were euthanized at the end of the study (14 days after RPXV inoculation). During the study, euthanasia decisions were made independently for each animal based upon three criteria: (1) severe respiratory distress as assessed by clinical observations including open mouth breathing, audible rales or wheezing, nasal flaring, or splinting, (2) moribund/persistent prostration and unresponsive to noxious stimuli, or (3) weight loss greater than 15% from the pre-challenge baseline weight. Animals requiring euthanasia were anesthetized with an IM injection of the ketamine/xylazine combination, and then administered an overdose of a euthanasia agent (Fatal Plus, Vortech Pharmaceuticals Ltd., Dearborn, MI) in accordance with the American Veterinary Medical Association euthanasia guidelines.

### Body temperature

During quarantine, each animal was sedated with acepromazine (IM; 2–3 mg/kg) and a temperature transponder chip (IPTT-300; BMDS, Seaford, DE) was implanted between the shoulder blades and at the rump area. For the natural history study, body temperatures were recorded twice daily at 12-h intervals (± 1 h) from Day−4 to 120 h post-challenge. After 120 h post-challenge, temperatures were recorded twice daily (AM and PM) until the end of the study. Body temperatures recorded from quarantine to Day 0 (AM) were used to establish a baseline. A hypothermic body temperature reading for the shoulder transponder chip of less than or equal to 37.2°C (99°F) was confirmed by a second reading approximately 1 h later.

Fever was defined as an observation of a greater than two standard deviation increase over pre-challenge mean baseline temperatures. The baseline standard deviation for each animal was calculated from pre-challenge temperatures for each study animal adjusted for rump/shoulder, AM/PM, and animal differences via an analysis of variance model. Time to fever (TTF) for each animal was defined as the time from challenge to the time the animal first had a temperature above the calculated fever threshold level.

### Blood collections

Blood samples were collected from the medial auricular artery or marginal ear vein daily and viral hemagglutinin (HA) gene copy number was determined by qPCR. Prior to blood collection, animals were sedated with acepromazine (IM; 2–3 mg/kg). Approximately 0.5 mL samples of whole blood were collected into EDTA tubes, and the DNA was extracted for qPCR.

### Viral burden by quantitative polymerase chain reaction (qPCR)

Total nucleic acid was isolated from whole blood samples (200 μL or the maximum sample volume available) using the Specific B protocol on a NucliSens® easyMAG™ instrument (BioMérieux, l′Etoile, France) according to the manufacturer's instructions. The final step of the isolation process was elution of total nucleic acid in 40 μL of NucliSens® easyMAG™ Extraction Buffer 3. Each nucleic acid sample was assayed in duplicate by qRT-PCR for detection of a portion of the hemagglutinin [HA(J7R)] gene using a 7900HT real-time PCR system (Applied Biosystems, Life Technologies Corp., Carlsbad, CA). Each 25 μL reaction contained 5 μL of purified total nucleic acid sample with the remaining volume consisting of TaqMan® Gene Expression Master Mix (2X, Applied Biosystems), sterile water, and a custom gene expression assay (Applied Biosystems) consisting of primers and a 3′-minor groove binding (MGB) probe specific for a portion of the HA(J7R) gene (Kulesh et al., 2004). Sequences for the primers and probe were: 5′-GATGATGCAACTCTATCATGTA-3′ (forward), 5′-GTATAATTATCAAAATACAAGACGTC-3′ (reverse), and 5′-FAM-AGTGCTTGGTATAAGGAG-MGBNFQ-3′ (probe). Thermal cycling conditions were: 50°C for 2 min, 95°C for 10 min, followed by 45 cycles of 95°C for 15 sec and 60°C for 1 min. A standard curve for qPCR was produced using a custom plasmid (GenScript USA Inc., Piscataway, NJ) containing a cloned insert of the primer and probe specific portion of the HA(J7R) gene. Ten-fold serial dilutions were performed on the plasmid DNA, resulting in a dilution range of 1 × 10^6^ to 0.1 copies/μL. The final concentration for each sample was reported as gene copies per milliliter of whole blood sample.

### Statistical analysis—lethality

Statistical analyses were performed separately for the 9-weeks and 16-weeks animals. The proportions of surviving animals were calculated for each group. If some animals survived the length of study, then pairwise two-sided Fisher's exact tests were performed to compare the proportions of surviving animals among the groups and probit regression was performed to identify the relationship between the base-10 log-transformed challenge dose and death. If there was a significant dose-response, then the LD_50_ was calculated.

The median survival times and 95% confidence intervals were calculated for each group. Kaplan-Meier curves providing the probability of survival beyond various study times were plotted for each group and pairwise log-rank tests were performed to compare the groups when survival time was considered in addition to overall survival. Bonferroni-Holm adjustments were made to maintain an overall 0.05 level of significance for the multiple pairwise comparisons performed.

### Statistical analysis –natural history

Statistical analyses were performed on each measured biomarker separately for the 9-weeks and 16-weeks animals. Furthermore, the analysis was performed combining the data across the two RPXV-infected groups within each animal age. The proportions of surviving animals were calculated for the RPXV-infected and sham-infected animals. A two-sided Fisher's exact test was performed to compare the proportion of surviving animals in the RPXV-infected group to that in the sham-infected group.

The median survival times and 95 percent confidence intervals were calculated for the RPXV-infected and sham-infected animals. Kaplan-Meier curves providing the probability of survival beyond various study times were plotted and a log-rank test was performed to compare the RPXV-infected animals to the sham-infected animals when the survival time was considered in addition to overall survival.

Lesions, viremia, and fever were evaluated to determine the time point that the animals became abnormal following RPXV-inoculation. For pox lesions, abnormal was defined as the first occurrence of lesions (back or mouth/nose/eye lesions). Similarly, for qPCR, viremia was defined as the first occurrence the HA gene was detected. Analyses for determining the onset of fever were performed separately for each transponder location in which fever was defined as a significant increased temperature (2 standard deviations) following challenge when compared with pre-challenge temperatures. The baseline standard deviation (0.97°F) was estimated from all pre-challenge temperatures, after adjusting for rump/shoulder, AM/PM, and animal differences, using an analysis of variance model. Time to fever for each animal was defined as the time from challenge that the animal first exhibited a temperature above a threshold level.

The proportions of abnormal animals were calculated for the RPXV-infected and sham-infected animals for each parameter. Median times to abnormal and 95 percent confidence intervals were calculated for the RPXV-infected and sham-infected animals for each parameter. Mean times to abnormal and standard deviations were also calculated for the RPXV-infected and sham-infected animals that became abnormal for each parameter.

To determine if the times to abnormal were significantly different between 9 and 16-weeks RPXV-infected animals, *t*-tests were performed for each parameter. A log-rank test was performed to determine if the times to death were significantly different between 9 and 16-weeks RPXV-infected animals.

## Results

### Lethality of rabbitpox virus in the NZW rabbit

Reports in the literature indicate that rabbitpox can lethally infect young rabbits via the intradermal route at low dose (Adams et al., 2007). The potency of the Working Virus Stock was measured by making dilutions to generate target virus challenge titers of 1,000, 300, 81, 27, 9, and 3 PFU. Back titers of the challenge doses were 900, 412, 74.0, 33.4, 8.66, and 4.66 PFU, consistent with the targeted dose. All 9-weeks group animals died prior to the end of the study (Table 1); therefore, pairwise Fisher's exact tests and the probit regression analyses were not performed. Since all animals died prior to the end of the study, the LD_50_ for the 9-weeks animals was estimated to be less than the lowest challenge dose of 4.66 PFU. Median survival times within the 9-weeks groups ranged from approximately 6.9 to 8.1 days post-challenge. Prior to adjusting for multiple comparisons among the 9-weeks animals, the survival times in the groups injected with challenge doses of 4.66, 8.66, and 412 PFU were significantly greater than the survival times in the group injected with a challenge dose of 900 PFU.

**Table 1:** Lethality for 9 and 16-weeks RPXV-infected rabbits in the LD_50_ study.

**Animal Age on Study Day 0**	**Target RPXV Dose (PFU)**	**Actual RPXV Dose (PFU)**	**Number Dead/Total**	**Median Time to Death in Days (95% CI)**
9-weeks	3	4.66	6/6	8.1 (6.9, 11.9)
	9	8.66	6/6	7.9 (6.9, 8.9)
	27	33.4	6/6	7.6 (6.9, 10.1)
	81	74.0	6/6	7.6 (6.2, 10.9)
	300	412	6/6	8.1 (6.9, 9.9)
	1000	900	6/6	6.9 (5.1, 8.2)
16-weeks	3	4.66	6/6	9.4 (7.9, 12.9)
	9	8.66	5/6	9.4 (7.9, –)
	27	33.4	6/6	9.0 (7.9, 10.1)
	81	74.0	4/6	11.0 (6.1, –)
	300	412	4/6	9.1 (6.9, –)
	1000	900	6/6	7.9 (6.9, 8.9)

*CI, Confidence interval*.

**Figure 1:**
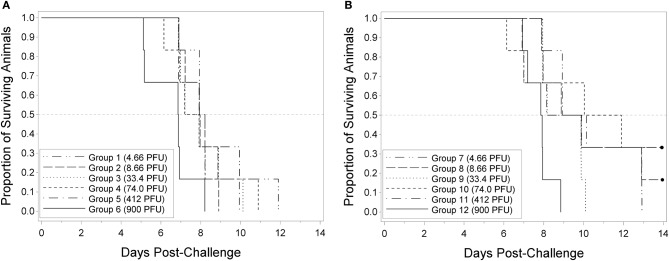
Kaplan-Meier curves representing survival times for 9-weeks **(A)** and 16-weeks **(B)** New Zealand White rabbits challenged by the intradermal route with RPXV in the LD_50_ study. Groups of 6 (3M/3F) rabbits for each age group were challenged at target doses of 3, 9, 27, 81, 300, and 1,000 PFU and followed for up to 14 days. Doses shown are the actual doses from the back titration.

All 16-weeks animals in the groups injected with challenge doses of 4.66, 33.4, and 900 PFU died prior to the end of the study. One animal survived in the group challenged at 8.66 PFU and two animals survived in each group challenged at 74.0 and 300 PFU. Regardless of the adjustment for multiple comparisons, there were no significant differences among the groups in terms of survival proportion. The probit regression resulted in a negative slope estimate that was not significantly different from zero; therefore, the LD_50_ could not be estimated. Median survival times within the 16-weeks groups ranged from approximately 7.9 to 11.0 days post-challenge. Prior to adjusting for multiple comparisons among the 16-weeks animals, the survival times in the groups injected with challenge doses of 4.66, 8.66, and 33.4 PFU were significantly greater than the survival times in the group injected with a challenge dose of 900 PFU. Figure 1 displays the Kaplan-Meier curves associated with survival times for the 9 and 16-weeks groups, respectively, for the lethality study. These results demonstrate that RPXV is lethal in both 9 and 16-weeks rabbits at low dose, similar to the lethality observed for variola virus in humans.

### Natural history of rabbitpox virus in NZW rabbit

**Figure 2:**
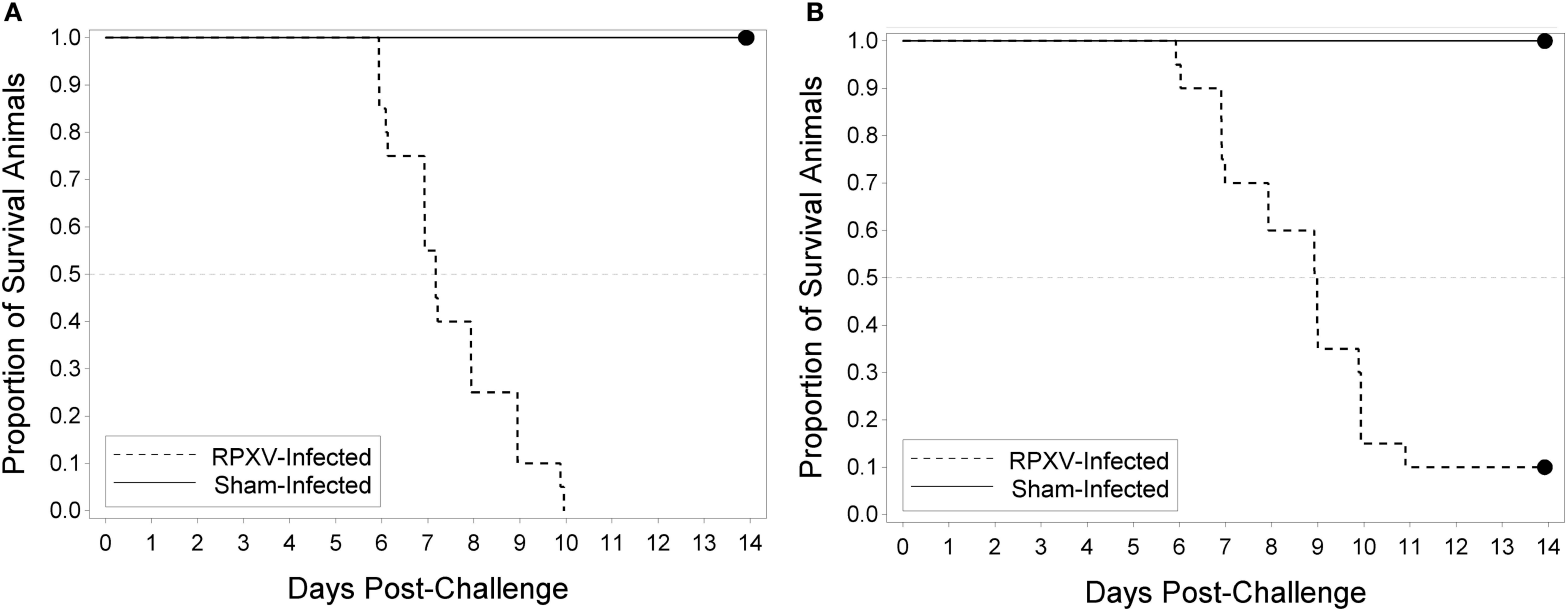
Kaplan-Meier curves representing survival times for 9-weeks **(A)** and 16-weeks **(B)** New Zealand White rabbits challenged by the intradermal route with RPXV in the natural history study. Twenty (10M/10F) rabbits for each age group were challenged with at target dose of 300 PFU (316 PFU actual) and followed for up to 14 days. The sham infected (DPBS) group consisted of 4 rabbits (2M/2F).

The utility of the RPXV challenge model as a tool for regulatory evaluation of anti-poxvirus products relies on the identification of biomarkers that can serve as triggers for medical intervention. These triggers should correlate with a specific stage in disease progression, need to be reproducible and unambiguous, and ideally reflect similar symptoms in human smallpox. Our approach toward identifying these triggers relied on the inclusion of sham infected animals as part of each experiment. Although infected and uninfected animals were housed separately, the clinical scoring and testing were performed by operators blinded to the disease state of the animal. Progression of RPXV infection in 9 and 16-weeks old rabbits was followed in a natural history study in which the rabbits were challenged with 300 PFU, a challenge dose predicted to result in >90% of the rabbits dying by day 11 post-challenge. The back titer of the challenge dose was 316 PFU per animal, consistent with the targeted dose. All twenty 9-week RPXV-infected animals died prior to the end of the study, while eighteen out of twenty (18/20) 16-weeks RPXV-infected animals died prior to the end of the study. All sham-infected animals (9 and 16-weeks) survived to the end of the study. A two-sided Fisher's exact test was performed to compare the proportion of surviving animals in the RPXV-infected group to that in the sham-infected group. For both the 9 and 16-weeks animals, the proportion of surviving sham-infected animals was significantly greater than the proportion of surviving RPXV-infected animals (*p* < 0.0001 and 0.0006 for 9 and 16-weeks, respectively). Figure 2 displays the Kaplan-Meier curves associated with survival times for the 9 and 16-weeks groups, respectively, for the natural history study.

In the 9-weeks RPXV-infected group, 3 animals were euthanized, 17 were found dead, and there were no survivors. In the 16-weeks RPXV-infected group, 7 animals were euthanized, 11 were found dead, and 2 survived to 14 days post RPXV-infection (end of the study). The two 16-weeks group animals that survived to the end of study also exhibited signs of disease consistent with RPXV infection. All sham-infected animals survived to the end of the study.

### Body temperature

**Figure 3:**
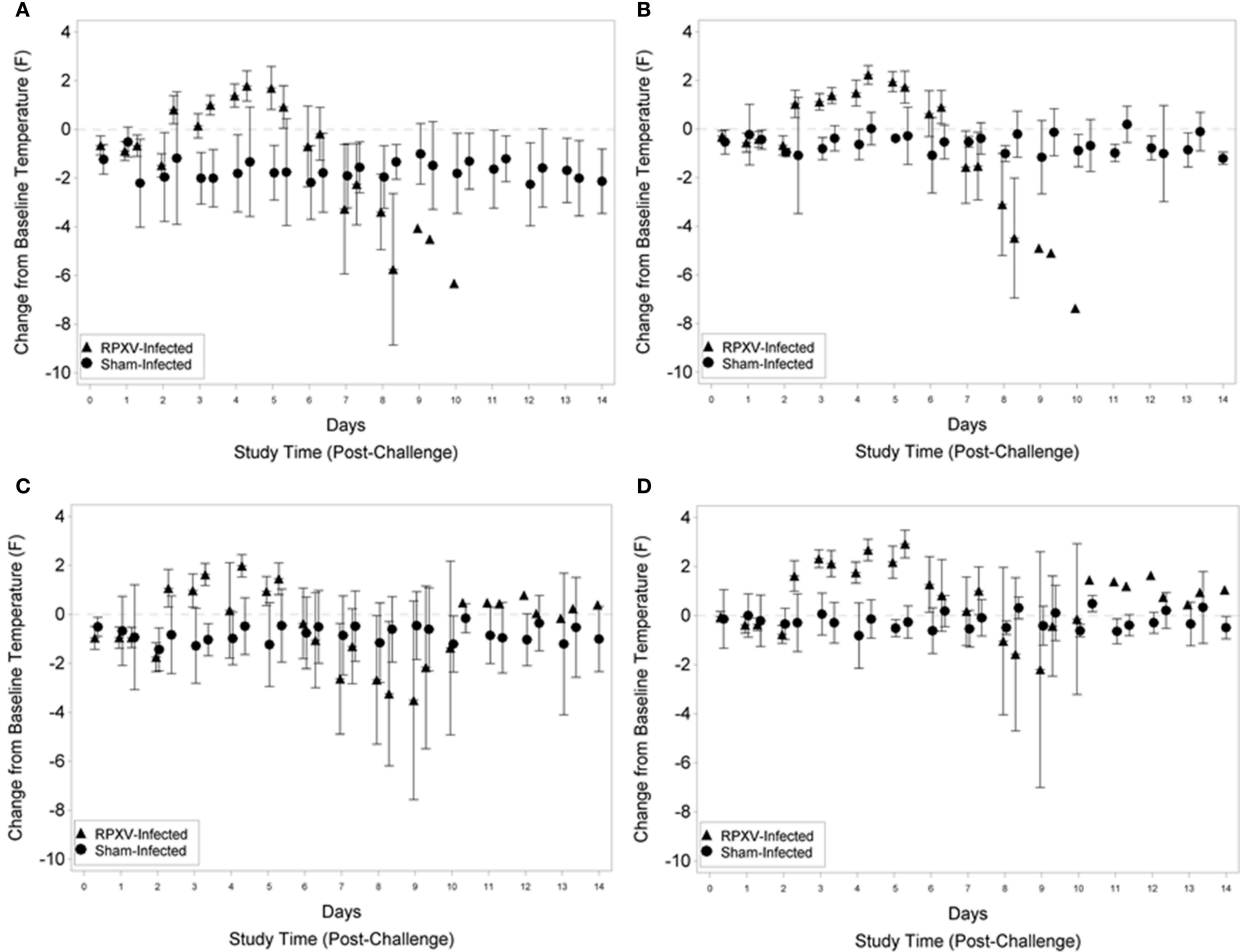
Group mean change from baseline temperature and 95 percent confidence intervals for 9-weeks **(A,B)** and 16-weeks **(C,D)** New Zealand White rabbits challenged by the intradermal route with RPXV at 316 PFU in the natural history study. The temperature transponder chip location was in the rump **(A,C)** or shoulder **(B,D)**. Confidence intervals were not displayed for the RPXV infected group at study times with three or fewer surviving animals.

For the 9-weeks animals, fever was first detected (rump and shoulder, Figures 3A,B, respectively) 2 days (PM) post RPXV-inoculation with the temperature remaining above baseline through 5 days (PM) post-RPXV inoculation. During this period, mean body temperatures were 0.15 to 1.79°F above baseline for the rump and 1.03 to 2.23°F above baseline for the shoulder. After 5 days post-RPXV inoculation, the mean body temperatures decreased and eventually the rabbits became hypothermic prior to death. Although some sham infected animals had occasions of slight temperature elevation compared to baseline, none of the sham infected animals had an increase in temperature that qualified as fever (2 standard deviations above baseline). The onset and frequency of fever were similar for the 16-weeks RPXV-infected group as for the younger rabbits. The first significant increase from baseline temperatures (rump and shoulder, Figures 3C,D, respectively) occurred 2 days (PM) post-RPXV inoculation with the temperature remaining above baseline through 5 days (PM) post-RPXV inoculation. During this period, mean body temperatures were 0.16 to 1.99°F above baseline for the rump and 1.27 to 2.92°F above baseline for the shoulder. From 5 to 9 days post-RPXV inoculation, the mean body temperatures decreased to below baseline, which coincided with most of the animal deaths. After 9 days post-RPXV inoculation, the mean body temperatures were still elevated above baseline for the few surviving animals.

### Viral burden

**Figure 4:**
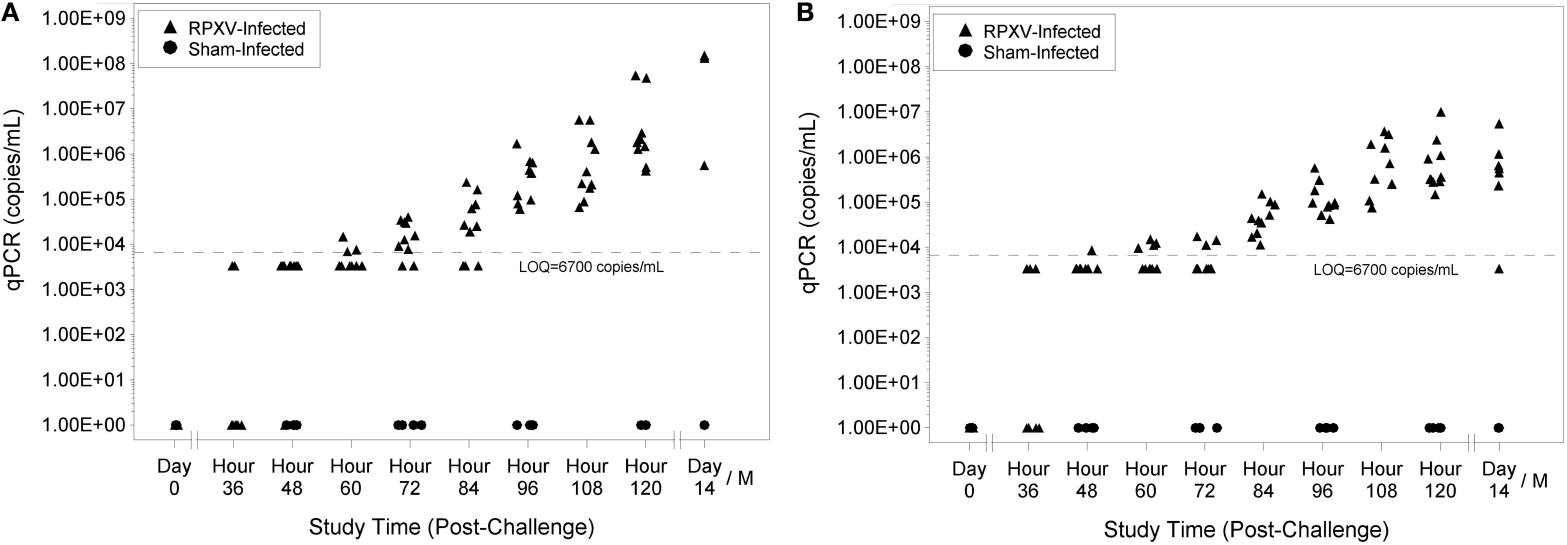
Blood qPCR levels for 9-weeks **(A)** and 16-weeks **(B)** New Zealand White rabbits challenged by the intradermal route with RPXV at 316 PFU in the natural history study.

Viral genome number was quantitated by qPCR analysis where each sample was run in duplicate and a quantifiable copy number and limit of quantitation (LOQ) was reported. Animals were considered viremic if their qPCR results had detectable levels of the HA gene (even if the copy number was detected in one sample). No sham-infected animals were viremic at any study time and no RPXV-infected animals were viremic prior to challenge. Nine of 10 of the 9-weeks RPXV-infected animals were viremic by 48 h post-RPXV inoculation and all were viremic by 60 h (Figure 4A). For the 16-weeks animals, 4 of 9 were viremic by 36 h post-RPXV inoculation and all were viremic by 48 h (Figure 4B). The level of RPXV HA gene copies increased temporally through the end of the study.

### Clinical signs and lesion count

All RPXV-infected animals exhibited adverse clinical signs of disease that included red swelling around the inoculation site, reduced food consumption, nasal and ocular discharge, and lethargy. The red swelling around the inoculation site was observed in several animals within the first 24 h and in 98% of the animals by 3 (PM) days post-RPXV inoculation. Inappetance was observed 6 days post-RPXV inoculation for all of the surviving infected animals. Sixty-three (63) percent of the rabbits developed nasal and ocular discharge, lethargy, mucosal stool, or reduced volume of stool 6 days post-RPXV inoculation. No abnormal observations were detected in sham infected rabbits with the exception of two animals exhibiting mucosal stool for less than a day, a single diarrhea observation, and one animal with a scab on its back.

**Figure 5:**
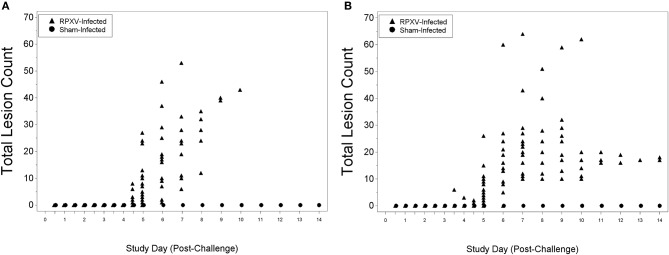
Total lesion count for 9-weeks **(A)** and 16-weeks **(B)** New Zealand White rabbits challenged by the intradermal route with RPXV at 316 PFU in the natural history study.

There were no lesions for all animals through 3 days (AM) post-RPXV inoculation. The 9-weeks animals began to display lesions at 4 days (PM) post-RPXV inoculation (Figure 5A), while the 16-weeks animals began to display lesions at 3 days (PM) post RPXV-inoculation (Figure 5B). Back lesions were more prevalent than mouth, nose, or eye lesions. No lesions were observed on sham infected animals.

### Time to abnormal

An analysis was performed to characterize the onset of disease symptoms in the RPXV-infected animals. Although a number of clinical signs such as inappetance, lacrimation, nasal discharge, and lethargy, were observed in the RPXV-infected animals, the signs occurred relatively late (initiating on or after 5 days post RPXV-inoculation), the signs were not universal in infected animals, and the subjective nature of these parameters, decreases their effectiveness as potential triggers for therapeutic intervention. RPXV-infected animals also exhibited weight loss after 5 days post-RPXV inoculation. For this reason, clinical signs and weight loss were not included in the statistical analysis for Time to Abnormal (TTA). The TTA results and median time to death for the 9 and 16-weeks group animals are provided in Tables 2, 3, respectively.

**Table 2:** Time to abnormal compared with time to death in 9-weeks RPXV-infected rabbits in the natural history study.

**Parameter**	**Number/Total**	**Median TTA/Death in Days (95% Confidence Interval)**	**Mean TTA in Days for Abnormal Parameter Only (*SD*)**
qPCR (Viremia)	20/20	2.00 (2.00, 2.50)	2.20 (0.38)
Temperature (Fever)	20/20	3.33 (3.00, 4.00)	3.68 (0.77)
Lesions	20/20	5.00 (4.50, 5.00)	4.93 (0.44)
Death	20/20	7.17 (6.13, 7.94)	NA

*TTA, Time to abnormal; SD, Standard deviation; NA Not available*.

**Table 3:** Time to abnormal compared with time to death in 16-weeks RPXV-infected rabbits in the natural history study.

**Parameter**	**Number/Total**	**Median TTA/Death in Days (95% Confidence Interval)**	**Mean TTA in Days for Abnormal Parameter Only (*SD*)**
qPCR (Viremia)	20/20	2.00 (2.00, 2.50)	2.05 (0.36)
Temperature (Fever)	20/20	3.00 (3.00, 3.33)	3.22 (0.49)
Lesions	20/20	5.00 (4.50, 5.00)	4.93 (0.61)
Death	18/20	8.96 (6.92, 9.93)	NA

*TTA, Time to abnormal; SD, Standard deviation; NA, Not available*.

All RPXV-infected animals developed fever, increased genome copy number, and lesions. None of the sham-infected animals developed these symptoms. The median time for viremia was 2 days post-RPXV infection for both the 9 and 16-weeks RPXV-infected animals. The median time to fever was 3.33 and 3.00 days for the 9 and 16-weeks rabbits, respectively. The median time for the presence of pox lesions was 5 days for both the 9 and 16-weeks RPXV-infected animals.

**Figure 6:**
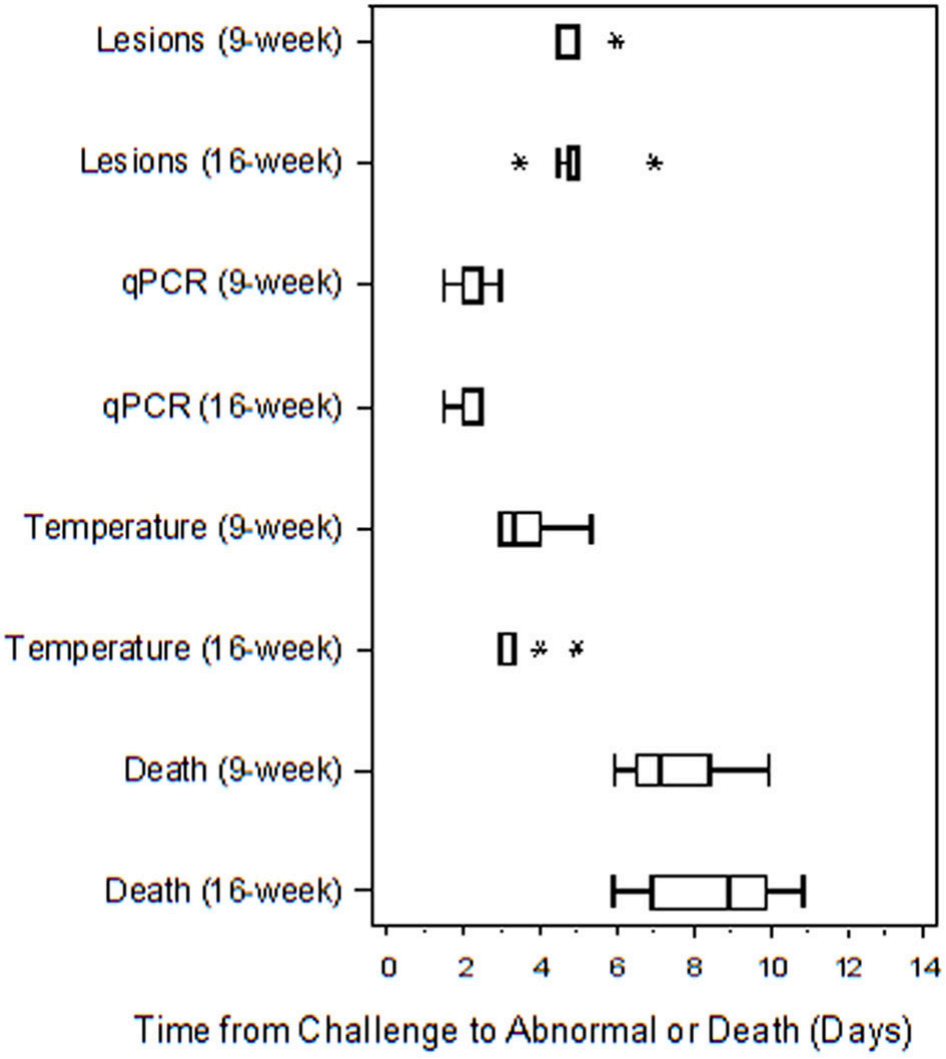
Boxplot of time to abnormal parameters or death for 9 and 16-weeks New Zealand White rabbits challenged by the intradermal route with RPXV at 316 PFU in the natural history study. The length of each box is equal to the interquartile range (IQR), defined as the difference between the 75 and 25th percentiles. The vertical line inside the box represents the median, while the horizontal lines to the left and right of the box extend to the minimum and maximum values that were not more than 1.5 times the IQR from the 25 and 75th percentiles. Values that were more than 1.5 times the IQR below the 25th percentile or 1.5 times the IQR above the 75th percentile are plotted with an asterisk.

Table 4 presents the results from the statistical analysis of the time to death and the TTA parameters between the 9 and the 16-weeks RPXV-infected animals. The mean time to fever among the 16-weeks RPXV-infected animals was significantly shorter than the time to fever in the 9-weeks RPXV-infected animals. There were no significant differences between the 9 and 16-weeks RPXV-infected rabbits with respect viremia, pox lesions, and time to death. The time course from RPXV-inoculation to abnormal for each parameter and death for the 9-week and 16-week rabbits is summarized in Table 5 and presented in Figure 6.

**Table 4:** Statistical significance of abnormal parameter and time to death in 9 and 16-Weeks RPXV-infected rabbits in the natural history study.

**Parameter**	***P*-value^a^**
qPCR (Viremia)	0.2054
Temperature (Fever)	0.0274^*^
Pox Lesions	1.0000
Death	0.1710

a *P-values for lesions, qPCR, and temperature are based on t-tests, while the p-value for death is based on a log-rank test*.

* *Significant at the 0.05 level*.

**Table 5:** Comparison of 9 and 16-weeks RPXV model parameters in the natural history study.

**Parameter**	**9-Weeks Rabbits**	**16-Weeks Rabbits**
Mortality at 316 PFU/animal	100%	90%
Median time to abnormal parameters	Viremic: Day 2.00 Fever: Day 3.33 Lesions: Day 5.00	Viremic: Day 2.00 Fever: Day 3.00 Lesions: Day 5.00
Median time to death	Day 7.17	Day 8.96
Maximum therapeutic window (days from viremic to death)	5.17 days	6.96 days

## Discussion

The possible introduction of smallpox into the U.S. population, either through natural or nefarious means, would be a high consequence event with the potential to kill millions of people. To mitigate the impact of such an event, the USG has invested money toward the development and licensure of medical countermeasures against smallpox. The portfolio of countermeasures under development includes vaccines and small molecules designed for prophylaxis and therapeutic use. In the absence of naturally-occurring smallpox disease, the evaluation of the medical countermeasures requires the use of animal models to demonstrate efficacy. The regulatory path for the evaluation of small molecule countermeasures in the U.S. utilizes the U.S. FDA Animal Rule (21 CFR 314.600 for drugs and 21 CFR 601.90 for biological products). The animal model used for the evaluation of medical countermeasure must address the intended use of the product, i.e., a prophylaxis countermeasure should be use a model where the product is administered prior to infection, while a therapeutic countermeasure must utilize a model with relevant triggers for medical intervention that correspond to symptoms in the human disease.

In this study, we demonstrated the adaptation and use of the RPXV intradermal challenge model for the therapeutic evaluation of smallpox countermeasures. The host restriction of VARV predicates the development and use of representative orthopoxvirus infections in animal models that recapitulate the steps in smallpox and provide predictive value as to the efficacy of countermeasures. The small animal models most commonly used in orthopoxvirus research are the mouse and rabbit (Chapman et al., 2010). The development of a rabbit model for product evaluation would be particularly beneficial as it combines reasonable blood volumes for disease monitoring and measuring product exposure and availability to design studies with statistical power. There are two rabbit infection models that appear to provide a reasonable recapitulation of smallpox in humans; the intradermal challenge model (Bedson and Duckworth, 1963; Adams et al., 2007) and the aerosol challenge model (Westwood et al., 1966; Nalca and Nichols, 2011). Both models have been used to investigate the efficacy of smallpox antivirals (Nalca et al., 2008; Roy and Voss, 2010; Rice et al., 2011a,b; Verreault et al., 2012; Trost et al., 2015; Grossi et al., 2017; Grosenbach et al., 2018). The evaluation of antivirals in these experiments relied on time-based treatment (post-exposure prophylaxis models) and not defined triggers for intervention resembling smallpox symptoms in humans.

The major advantage of an aerosol challenge model is that it exposes the animal via the same respiratory route that smallpox is transmitted in humans. However, we chose to concentrate on the development of an intradermal challenge model for a number of reasons including: (1) High mortality: Although high mortality can be achieved using either the intradermal or aerosol challenge routes, the aerosol route exhibits variability in mortality at low doses due to the uncertainty associated with delivering and measuring a low dose aerosol exposure and extensive bronchopneumonia at high doses. The intradermal route consistently establishes a “smallpox-like” disease of uniform mortality at low doses; (2) Reproducibility: An intradermal challenge ensures that every animal receives an identical dose, resulting in a synchronous disease. This provides a more evaluable approach for the testing of medical countermeasures; (3) Ease of use: The intradermal challenge route is easy, requires no specialized training or equipment and can be performed at all non-clinical labs; (4) Scalability: An advantage to the intradermal challenge route is the ability to easily execute experiments requiring large group sizes. The ability to do large group sizes is essential for ensuring studies possess sufficient statistical power to satisfy regulatory authorities and to permit studies such as comparative efficacy studies between countermeasures to be executed within reasonable timelines; (5) Disease progression: The results from Adams et al. (2007) suggested that rabbitpox disease in rabbits closely mimics the steps of smallpox disease in humans. After the initial infection, there is a symptom free incubation period, followed by fever and the dissemination of virus in the blood and the establishment of a secondary infection, evinced by disseminated lesions, followed by death. The infections established after intradermal or aerosol exposure results in a disease that is transmissible (Bedson and Duckworth, 1963; Westwood et al., 1966; Adams et al., 2007); and (6) Regulatory advice: The goal of this study was to establish a platform for the evaluation of medical countermeasures against smallpox. These studies were submitted to the FDA, and some elements of the protocol have been included at their suggestion, including the route and dose of challenge.

These studies were intended to determine if the intradermal challenge model is a platform for the evaluation of medical countermeasures, and in particular, therapeutic countermeasures against smallpox. In contrast to evaluation of prophylactic treatments, such as vaccines, the evaluation of therapeutic countermeasures does not rely on the challenge route as long as the disease at the time of intervention resembles smallpox strongly enough to provide evidence supporting the analogous activity of the countermeasure against smallpox. The important components of a model for regulatory evaluation include: (1) High mortality. In order to increase confidence in the efficacy of drugs that can only be evaluated using the Animal Rule, models are used that yield high mortality, representing the most stringent of challenges. This also simplifies statistical analysis and reduces the total number of animals required per study; (2) Scalability and Reproducibility. Studies for regulatory evaluation of products require large enough group sizes to ensure statistical significance and universality in protocol and equipment to allow the studies to be reproduced by many labs; and (3) Triggers for medical intervention. The disease in the animal model should elicit symptoms that are unambiguous and reproducible for medical intervention. It is most useful if these symptoms are similar or can be bridged to symptoms observed in human smallpox. Other characteristics, such as secondary endpoints for confirmatory evaluation of countermeasure effectiveness, animal availability, and model robustness also contribute to the value of an animal model.

Our general approach was to demonstrate lethality in the RPXV intradermal challenge route, choose a challenge dose that would provide high mortality, and to monitor the onset of biomarkers that are reproducible, unambiguous, and if possible, objective indications of disease. To ensure that biomarkers would correspond to disease, sham-infected animals were included in the natural history experiments to reduce the possibility of pursuing ambiguous or subjective markers. It is also important that the biomarkers chosen represent stages of the disease that can be correlated with smallpox in humans. The natural history experiments showed that RPXV disease in rabbits was analogous to smallpox in humans as indicated by a symptom-free prodromal period with no detectable circulating virus, followed by fever with coincident systemic dissemination of virus through the bloodstream, subsequent secondary infections manifested as lesions, and either death or resolution of the disease. The primary difference between RPXV in rabbits and smallpox in humans is that disease progression is compressed temporally in the rabbit model.

The most obvious clinical sign for smallpox infection is the synchronous peripherally-weighted rash of lesions that is eponymous for the disease. Detectable lesions have been reported as a consequence of RPXV infection within 5 days post-infection, especially on shaved backs, and as early as 3 days post-exposure presenting as lesions within the vasculature of the rabbit ear (Adams et al., 2007). Identification of lesions as a trigger for treatment would provide a clear and obvious bridge to the human symptoms for the animal model. Although ear lesions are not generally observed in human smallpox, the onset of this biomarker in rabbits potentially provided an early window for product intervention.

The development of a small animal model for therapeutic evaluation of smallpox countermeasures would provide the ability to perform studies measuring reasonable putative triggers for treatment. Although there may be measurable changes in clinical chemistry or virus detectable in internal organs during the disease, the labor required to confirm these biomarkers, make them less amenable to studies with large group sizes. The studies described here concentrated on three practical markers, including body temperature, viremia by qPCR, and lesions. These are clinically-relevant markers as temperature is easily measured in the field, the diagnostic test for smallpox presently deployed relies on qPCR analysis, and lesions are already considered confirmatory of smallpox infection.

The onset of these three markers was reproducible, unambiguous and synchronous in both 9 and 16-weeks old rabbits after RPXV infection. The detection of circulating viral genomes was observed at approximately 60 h post-exposure, followed by fever at 72 h post-exposure and secondary disseminated lesions approximately 2 days later. These markers provide clinical analogs to the expected operational response to a smallpox emergency. Although the index cases may only be identified upon lesion formation, it is reasonable to conclude that subsequent suspected cases will be treated at detection of viral copies, or upon fever.

One observation during the course of the model development studies was that the health of 9-weeks old rabbits was more tenuous compared to rabbits a few weeks older and more robust. Therefore, in order to test for a more humane and reproducible model, we measured the onset of these biomarkers in 16-weeks old rabbits to explore the robustness of the model. The onset of symptoms was coincident with observations for the 9-weeks rabbit. The only differences between the 9 and 16-weeks rabbits were that the mortality was less than 100% at the challenge dose of 300 PFU and the mean time to death was delayed in the older rabbits. The LD50 could not be calculated since the challenge doses were not high to give 100% mortality in some of the higher challenge groups.The estimated LD_50_ provided sufficient information to allow the designation of a dose that would be100% lethal when the model was used to evaluate medical countermeasures.

The second small animal model that CDER posited for evaluation of medical countermeasures against smallpox, the ectromelia mouse challenge model was described previously (Garver et al., 2016). In this model, BALB/c mice were challenged by the intranasal route with ectromelia, strain Moscow. Both the mouse and rabbit small animal models demonstrated universal mortality with a compressed 9 to 10-days disease course compared to the 2 to 3 weeks seen in human smallpox. In both models, molecular evidence for infection by qPCR and plaque assay preceded death, although in the mouse model detection, of viral genomes in the blood qPCR was later in the course of infection than for the rabbit. In the mouse model, overt signs of infection such as weight loss, temperature change, and clinical signs were only observed just prior to euthanasia. The course of the disease in the rabbit model appears to be more analogous to human smallpox in that fever is followed by detection of viral genome copies in the blood, followed by a disseminated lesional rash indicative of systemic infection, and subsequently by death. In the mouse model, the virus is detected early in the liver and spleen, followed by evidence for virus in the blood and death shortly thereafter. The rapid mortality after detection of virus in the blood in the mouse model appears to preclude the development of a lesional rash symptomatic of human smallpox.

The combination of these two models provides drug developers with useful tools for product evaluation. The primary model in use for regulatory evaluation of smallpox countermeasures, the intravenous monkeypox challenge model is hampered by the technical restrictions on group size in using non-human primates and facilities since it is restricted to BSL-3. The rabbit model, and the mouse model described earlier, can be carried out at BSL-2 using large enough group sizes to provide statistical power. The rabbit model also has the advantage of larger size and greater blood volume allowing for venous access ports for pharmacokinetic studies or slow intravenous infusions.

These results demonstrate the applicability of the RPXV infection model of rabbits as a suitable therapeutic model for the evaluation of smallpox countermeasures. The uniformity and timing of the biomarkers described in this manuscript provide reliable and relevant points for medical intervention with potential smallpox countermeasures to aid in their efficacy evaluation under the FDA Animal Rule.

The results of these experiments were presented to the FDA under a pre-IND to solicit guidance for the use of this model as a therapeutic model for product evaluation. Some of the experimental protocols for the natural history studies were adopted after FDA consultation. The FDA also suggested the derivation of a clonal isolate of RPXV would be an appropriate challenge virus for medical countermeasure evaluation. Subsequently, a clonal isolate was derived from three rounds of plaque purification on CV-1 cells, expanded through infection of CV-1 cells, purified by centrifugation through sucrose and stored as aliquots. This RPXV clonal isolate was shown to be free of adventitious agents, sterile, and exhibited 100% homology to the RPXV nucleotide sequence of the HA gene. The virus was equally virulent and elicited the same set of symptoms as the stock used in this manuscript. The RPXV infection model has been used for countermeasure efficacy studies including a recent smallpox disease treatment that was approved by the FDA (Grosenbach et al., 2018; https://www.fda.gov/downloads/advisorycommittees/committeesmeetingmaterials/drugs/anti-infectivedrugsadvisorycommittee/ucm605890.pdf).

## Author contributions

MP, RW, MM, and CH: designed the studies; MP performed studies; MP: analyzed data; All authors wrote the manuscript.

### Conflict of interest statement

The authors declare that the research was conducted in the absence of any commercial or financial relationships that could be construed as a potential conflict of interest.
